# Women’s mental health during the COVID 19 pandemic and the problem of domestic violence in a lockdown situation

**DOI:** 10.1192/j.eurpsy.2022.2237

**Published:** 2022-09-01

**Authors:** M. Kachaeva, N. Semenova, S. Shport, V. Vasyanina, O. Shishkina, N. Skibina, L. Nazarova

**Affiliations:** 1 V. Serbsky National Medical Research Centre for Psychiatry and Narcology, Forensic Psychiatry, Moscow, Russian Federation; 2 Moscow Research Institute of Psychiatry, Clinical Psychology Counseling, Moscow, Russian Federation; 3 V. Serbsky National Medical Research Centre for Psychiatry and Narcology, Moscow Research Institute Of Psychiatry, Moscow, Russian Federation; 4 I.M.Sechenov First Moscow State Medical University, Forensic Psychiatry, Moscow, Russian Federation

**Keywords:** women, women, mental health

## Abstract

**Introduction:**

The current situation caused by restrictive measures related to the COVID-19 pandemic provokes the high level of aggressiveness and all forms of domestic violence. These results in mental health problems.

**Objectives:**

The purpose of this study was to find out the consequences of domestic violence against women and to identify psychological problems and mental disorders in women.

**Methods:**

A cohort of 18 females was examined by psychiatrists and psychologists. All women turned to specialists at the Moscow Institute of Psychiatry for help.

**Results:**

All women were victims of violence by their husbands and partners. Lockdown situation associated with COVID 19 pandemic has exacerbated the problem of domestic violence. Clinical assessment has revealed different depressive symptoms, anxiety, fear, suicidal tendencies forming the clinical picture of adjustment disorder (2 cases), acute reaction to stress (3 cases), post-traumatic stress disorder (2 cases), depressive episode (8 cases), eating disorders behavior in the form of bulimia and anorexia (3 cases).

**Conclusions:**

The research has revealed that domestic abuse against women associated with lockdown situation during the COVID 19 pandemic often results in psychological and long-term mental health problems. In these cases, prevention is needed to combat violence against women with the participation of public health specialists, psychologists, psychiatrists, sociologists.

**Disclosure:**

No significant relationships.

